# Radiologic approach to axial spondyloarthritis: where are we now and where are we heading?

**DOI:** 10.1007/s00296-018-4130-1

**Published:** 2018-08-21

**Authors:** Iwona Kucybała, Andrzej Urbanik, Wadim Wojciechowski

**Affiliations:** 1 Chair of Radiology, Jagiellonian University Medical College, 19 Kopernika Street, 31-501 Krakόw, Poland; 2 Comarch Healthcare S.A., 29 Życzkowskiego Street, 31-864 Krakόw, Poland

**Keywords:** Sacroiliac joints, Axial spondyloarthropathy, Ankylosing spondylitis, EULAR recommendations, ASAS recommendations, Magnetic resonance imaging

## Abstract

Current emphasis on diagnosing axial spondyloarthritis (axSpA) in early stage enforced the search for sensitive and specific diagnostic algorithms with the use of imaging methods. The aim of this review was to summarise current recommendations concerning the use of imaging techniques in diagnostics and monitoring of axSpA as well as to outline possible future directions of the development in this field. MEDLINE database was searched between March and April 2018. In the first phase, such keywords were applied: ‘ASAS’, ‘EULAR’, ‘ASAS-EULAR’, ‘ASAS/OMERACT’, ‘axial spondyloarthritis’, while in the second step: ‘axial spondyloarthritis’, ‘ankylosing spondylitis’, ‘magnetic resonance imaging’, ‘computed tomography’, and ‘radiography’, ‘imaging’. An up-to-date summary of European League Against Rheumatism (EULAR) recommendations enriched with recent updates of Assessment of Spondyloarthritis International Society (ASAS) diagnostic criteria regarding imaging in axSpA course was created. Moreover, we outlined the role of new in this field, promising imaging techniques, such as diffusion-weighted imaging and dynamic contrast-enhanced sequences in magnetic resonance imaging (MRI) or low-dose computed tomography (CT). As precise monitoring of axSpA activity is vital, we reviewed the most precise methods: semiquantitative scores (e.g., Spondyloarthritis Research Consortium of Canada scores or CT Syndesmophyte Score) and quantitative analysis of MRI-based apparent diffusion coefficient or perfusion maps and enhancement curves. According to EULAR and ASAS recommendations, radiography and MRI still remain basic methods of axSpA diagnostics and monitoring. However, the knowledge of state-of-the-art international guidelines combined with the awareness of emerging imaging methods is the key to effective management of axSpA.

## Introduction

The concept of ‘spondyloarthritis’ describes a heterogeneous group of chronic inflammatory rheumatic diseases, which subdivides into two categories: axial (axSpA) and peripheral (pSpA) spondyloarthropathies [1, 2]. The former group, axSpA, gathers broader spectrum of states involving the sacroiliac joints (SIJs) and the spine—ankylosing spondylitis (AS) and non-radiographic axSpA [3].

Since the major symptom of the axSpA is chronic back pain, which is highly prevalent in population and not specific, it became obvious that definite diagnostic criteria had to be established [3]. Apart from clinical symptoms, radiological findings have been integral part of the AS diagnosis since the 1930s—especially detected on the SIJs radiography, as it is the point of disease origin in almost all cases of AS. The first official set of AS diagnostic criteria, which included radiological assessment of the SIJs, was the modified New York (mNY) criteria, published in 1984. Later on, the Amor (1990) and European Spondyloarthropathy Study Group criteria (1991), created for the diagnosis of spondyloarthropathies in general, contained the same definition of sacroiliitis as mNY criteria [4].

Nonetheless, it was the Assessment of Spondyloarthritis International Society (ASAS) criteria for axSpA (2009) which triggered real breakthrough in diagnostics of this disabling condition. The creation of separate diagnostic arm based on the radiological visualisation of sacroiliitis and incorporating magnetic resonance imaging (MRI) as a sufficient method of SIJ inflammation detection, put enormous emphasis on the importance of radiology in the diagnosis of axSpA [5]. Capturing the disease on early, pre-radiographical stage, what became possible owing to MRI, is especially relevant in term of quick implementation of effective therapy—namely, TNF inhibitors, which are approved in Europe also for non-radiographic axSpA [6].

On the other hand, a lot of authors criticise the ASAS criteria [6–9], predominantly because of heterogeneity in clinical characteristics and response to TNF inhibitors between populations diagnosed by imaging and clinical arms [8]. Another raised question is a tendency to lower specificity of imaging arm in previously unselected population with chronic back pain, as lesions of mechanical origin may mimic changes characteristic for axSpA on MRI [9]. Thus, staying up-to-date with current recommendations of international societies (e. g., European League Against Rheumatism—EULAR, ASAS) and regular search for more and more specific methods of axSpA evaluation is crucial for physicians dealing with this subject professionally.

The aim of this review was to summarise current recommendations regarding the use of imaging methods in diagnostics and monitoring of axSpA and to outline possible future directions of development in this field which may improve an effectiveness of early axSpA detection.

## Search strategy

A comprehensive search of the MEDLINE database was conducted in the period between March and April 2018. The final search was carried out on 6 April 2018. Only published data written in English was taken into account. In the first phase of the search, such keywords were applied as ‘ASAS’ or ‘EULAR’ or ‘ASAS-EULAR’ or ‘ASAS/OMERACT’ and ‘axial spondyloarthritis’ to obtain only up-to-date recommendations of international societies regarding the use of imaging in diagnosis and management of axial spondyloarthritis. Preference was given to the sources published within the past 9 years. In the next step, the search was conducted with the use of the following keywords: ‘axial spondyloarthritis’ or ‘ankylosing spondylitis’ and ‘magnetic resonance imaging’ or ‘computed tomography’ or ‘radiography’ or ‘imaging’. Titles and abstracts were analysed to identify articles covering the topic of promising imaging methods which have not been investigated thoroughly yet in the field of axSpA as well as quantitative and semiquantitative methods of axSpA assessment with the use of imaging. In this case, preference was given to articles published since 2005. In addition, reference lists of articles which met inclusion criteria in both cases were screened for other eligible studies.

## Assessment of the sacroiliac joints

### Conventional radiography (CR)

According to EULAR recommendations (2015) [1]:

1. CR of the SIJs is advised as the first imaging technique to diagnose sacroiliitis associated with axSpA.
2. CR of the SIJs may be used for long-term structural damage monitoring in axSpA—especially new bone formation.

The radiographic definition of axSpA-related sacroiliitis currently used in EULAR recommendations and the imaging arm of ASAS axSpA diagnostic criteria, which is equal to mNY criteria, is [5]:

1. Bilateral sacroiliitis grade ≥ II.
2. Unilateral sacroiliitis grade ≥ III.

Details regarding particular grades are shown in Table 1 [4].

**Table 1:** Grading of radiographic sacroiliitis

Grade	Stage	Explanation
0	Normal	Unchanged morphology of the joint
I	Suspicious	Blurring of the joint margins
II	Minimal abnormality	Small localised areas of erosions or sclerosis, without alteration of the joint width
III	Unequivocal abnormality	Moderate/advanced sacroiliitis: ≥ 1 out of: erosions, sclerosis, widening/narrowing of the joint space, partial ankylosis
IV	Severe abnormality	Total ankylosis

The primary drawback of CR in the assessment of SIJs is that it enables to visualise only late, post-inflammatory changes—capturing the disease on such advanced stage may significantly diminish the positive effect of biologic therapy, which is effective only with coexisting acute inflammatory changes. Moreover, due to the complex anatomy of the SIJs, multiple techniques of their visualisation and highly subjective classification of the sacroiliitis signs, the reliability of this method in less evident cases is arguable. However, up to this point, any other imaging method has not surpassed the CR in terms of cost-effectiveness, widespread availability, and relatively low dose of ionizing radiation. Hence, the CR most likely remains the basic modality used for sacroiliitis detection in forthcoming years [10]. There may only be an attempt made to implement more objective radiographic classification of the SIJs structural damage than existing mNY criteria.

### Computed tomography (CT)

According to EULAR recommendations (2015) [1]:

1. CT might provide additional information on structural damage if CR is negative and MRI contraindicated or cannot be performed.

CT is regarded as a gold standard of the SIJs structural damage detection [11]. Nonetheless, the most important sign of active inflammation of the SIJs—bone-marrow oedema—is not visible on this imaging modality. The same applies to fatty degeneration of bone marrow, which is an indicator of the early phase of chronic joint inflammation [4]. Combining aforementioned difficulties with high dose of ionizing radiation and relatively high costs, when compared to CR, reluctance towards CT use in diagnostics of axSpA does not come as a surprise. On the other hand, low-dose CT of the SIJs seems to be very promising technique and may have a chance to replace CR as a method of structural damage and new bone formation monitoring [12, 13]. Another promising method is spectral CT, which enables to measure both calcium and water concentration within a tissue. As a consequence, the visualisation and quantitative analyses of the bone-marrow oedema within the SIJs are possible and this method may be a leap forward more accurate diagnosis of the axSpA [14, 15].

### Magnetic resonance imaging

According to EULAR recommendations [1]:

1. In some cases, such as young age of the patient or short symptom duration, MRI of the SIJs is an alternative to CR first imaging method of axSpA diagnosis.
2. When the diagnosis of axSpA cannot be established based on clinical features along with CR and axSpA is still suspected, MRI of the SIJs is recommended. Consider the presence of both active inflammatory lesions (bone-marrow oedema) and structural lesions (bone erosion, new bone formation, sclerosis, and fat infiltration).
3. MRI of the SIJs may be used to assess and monitor disease activity in axSpA providing additional information to clinical and biochemical assessments. The decision regarding repeating MRI depends on the clinical circumstances. Short tau inversion recovery (STIR) sequences are sufficient to detect inflammation.
4. MRI of the SIJs may provide additional information in monitoring of their structural changes.
5. Extensive MRI inflammatory activity (bone-marrow oedema) might be used as a predictor of good clinical response to anti-TNFα treatment in axSpA. Hence, MRI might aid in the decision of initiating biologic therapy, in addition to clinical features and CRP.

**Fig. 1:**
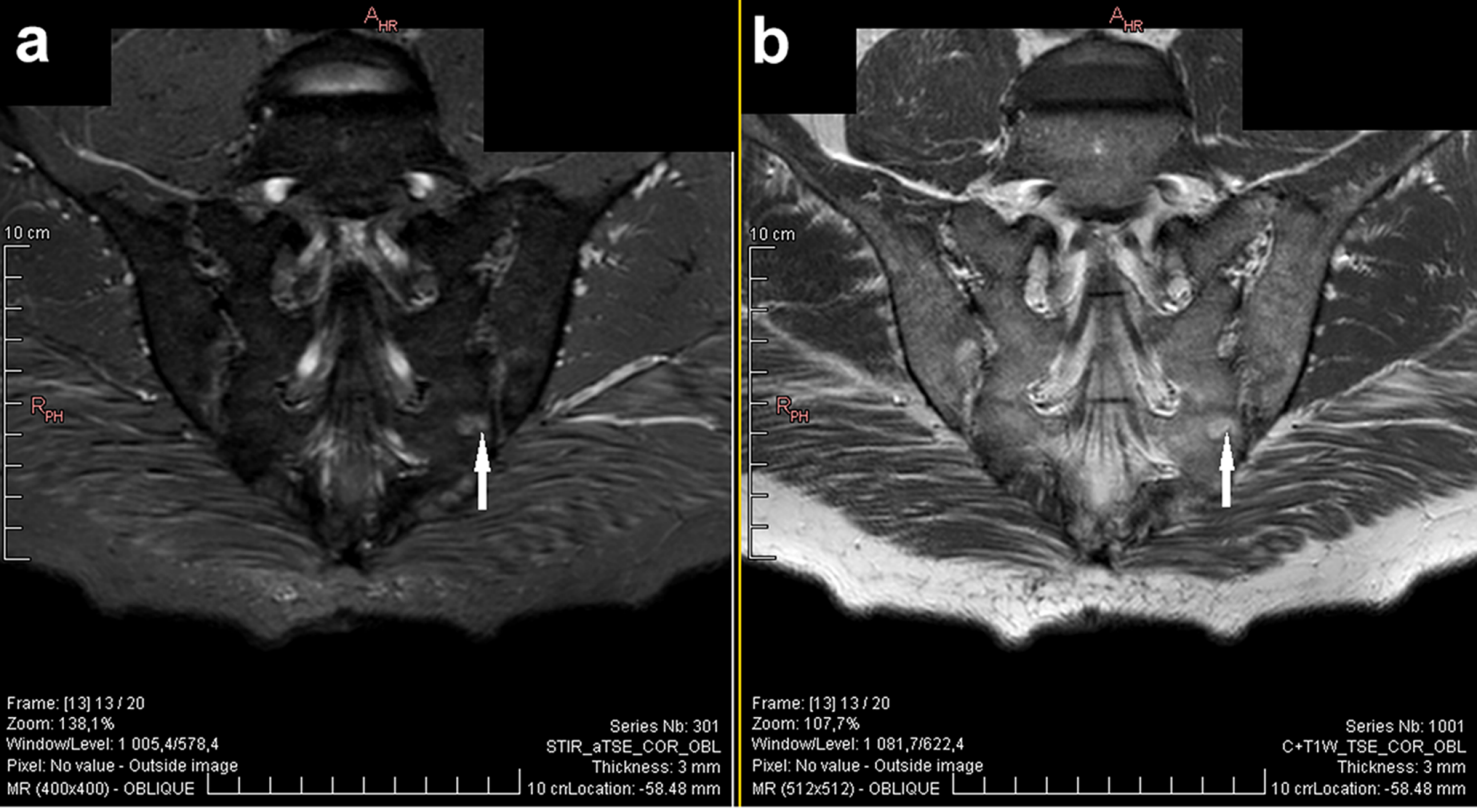
Typical axSpA bone-marrow oedema lesion (white arrow) in the lower sacral quadrant of the left sacroiliac joint on STIR sequence (**a**) and contrast-enhanced T1-weighted sequence (**b**). STIR, short tau inversion recovery

As far as axSpA diagnosis is concerned, the imaging arm of the ASAS criteria as one of the two equivalent definitions of sacroiliitis includes signs of acute inflammation highly suggestive of axSpA on MRI of the SIJs [5]. The latest update of the definition of active sacroiliitis in the course of axSpA by the ASAS MRI working group (2015) equate active inflammation on MRI only with bone-marrow oedema/osteitis and did not extend the definition of other active inflammatory lesions (such as capsulitis, synovitis, and enthesitis) or signs of chronic inflammatory changes (such as erosions, sclerosis, fat deposition, and bony bridges/ankylosis) [4, 16]. Precise definition of bone-marrow oedema according to aforementioned criteria is a presence of bone-marrow lesion, which is hyperintense on water-sensitive T2-weighted sequences (e.g., STIR or fat-suppressed T2-weighted) or enhancing on T1-weighted sequences after the contrast media administration. If one lesion is present, it must be visible on two consecutive slices of an MRI scan, while the presence of at least two lesions on one slice is sufficient to diagnose sacroiliitis. As well, the lesion must be located in the subchondral bone in the region adjacent to articular surface of the SIJs [16]. An example of typical bone-marrow oedema lesion in the SIJs is shown in Fig. 1.

Although some authors proposed the inclusion of structural lesions to the definition of sacroiliitis highly suggestive of axSpA [17, 18], there were too many discrepancies with regard to a precise definition of these structural changes appearance on MRI to include them in the final version of the updated sacroiliitis definition. Nonetheless, in cases when it is questionable if lesions visualised on the SIJs MRI are “highly suggestive of axSpA”, the evaluation of structural changes within the SIJs (especially erosions) might be beneficial for reaching the final diagnosis [16].

There exists a wide range of scoring systems facilitating quantitative evaluation of active sacroiliitis on MRI, but the best performing one is the Spondyloarthritis Research Consortium of Canada (SPARCC) score [19], proposed by Maksymowych et al. [20]. This method is based on the evaluation on STIR sequence six consecutive semicoronal slices focusing on the synovial part of the SIJ, in the direction from posterior to anterior. Each SIJ is divided into four quadrants (upper iliac, lower iliac, upper sacral, and lower sacral) and every one of them is separately assessed. First, each quadrant is analysed for the presence of the hyperintense lesions in STIR sequence and scored dichotomously (0 = normal signal, 1 = present lesion of increased intensity). Each quadrant could also receive an additional one point for the presence of intense signal within the lesion, and the next one for continuously increased signal for ≥ 1 cm from the articular surface. Summing it up, every slice maximally could get 12 points, what gives 72 points for all six slices [20]. Although assessment of the chronic inflammatory changes is not mandatory to diagnose axSpA, it is still worth performing their quantitative evaluation even for purposes of more objective disease monitoring. Analogous to previously mentioned SPARCC score for active inflammatory changes, the SPARCC MRI sacroiliac joint structural score (SSS) enables to appraise the presence of fat metaplasia, erosion, backfill, and ankylosis [21].

**Fig. 2:**
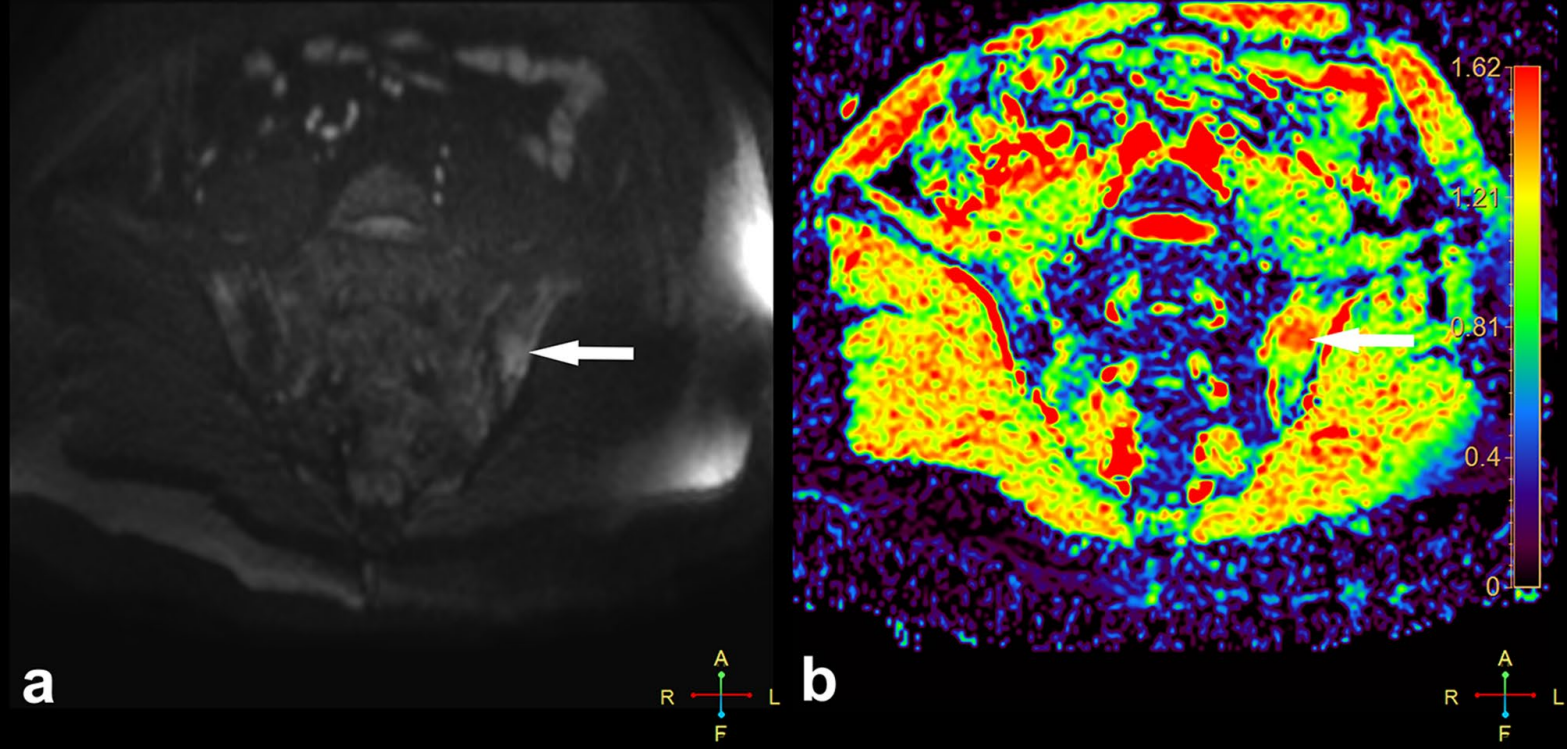
Active inflammatory lesion in the course of axSpA (white arrow) located in the iliac part of the left sacroiliac joint on DWI (*b* = 800) sequence (**a**) and colour ADC map (**b**). DWI, diffusion-weighted imaging; ADC, apparent diffusion coefficient

**Fig. 3:**
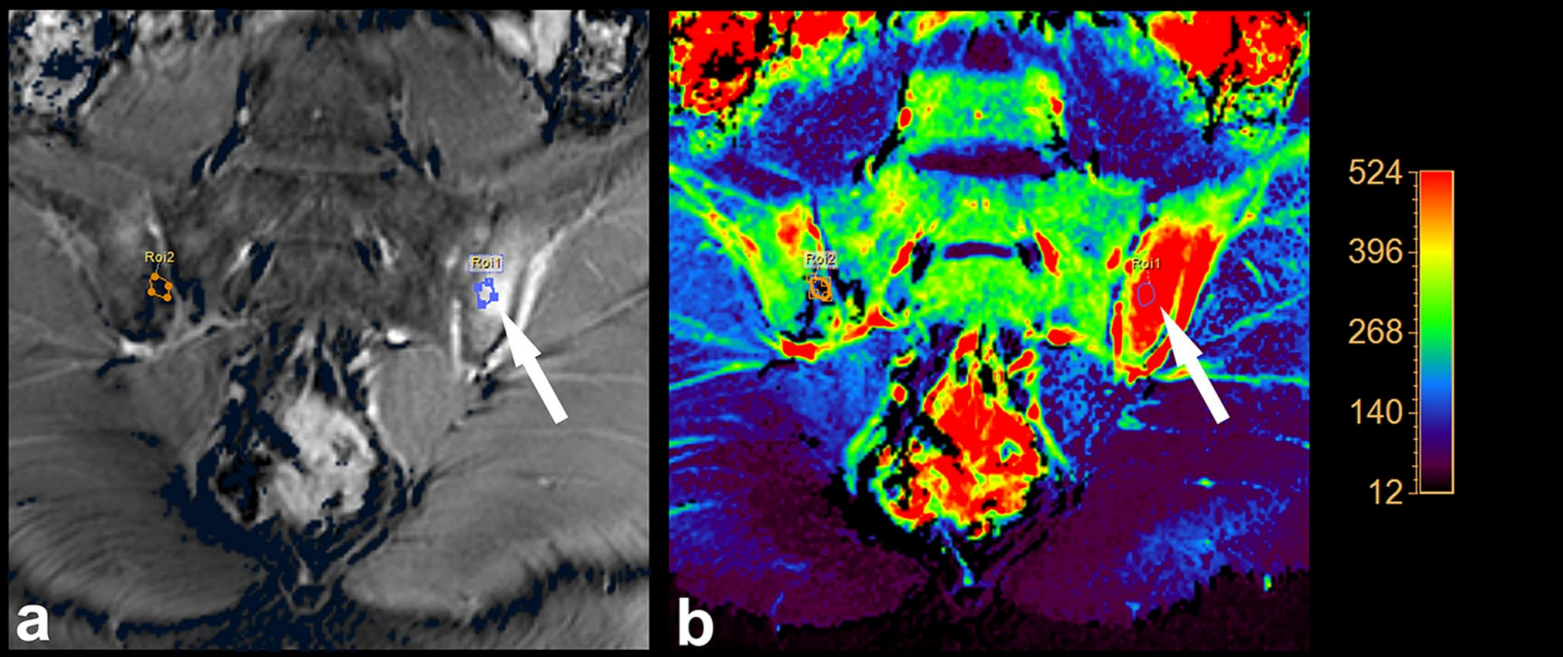
Active inflammatory lesion in the course of axSpA (white arrow) situated in the iliac part of the left sacroiliac joint visualised with use of DCE sequence (**a**) and maximal perfusion colour-coded map (**b**)

**Fig. 4:**
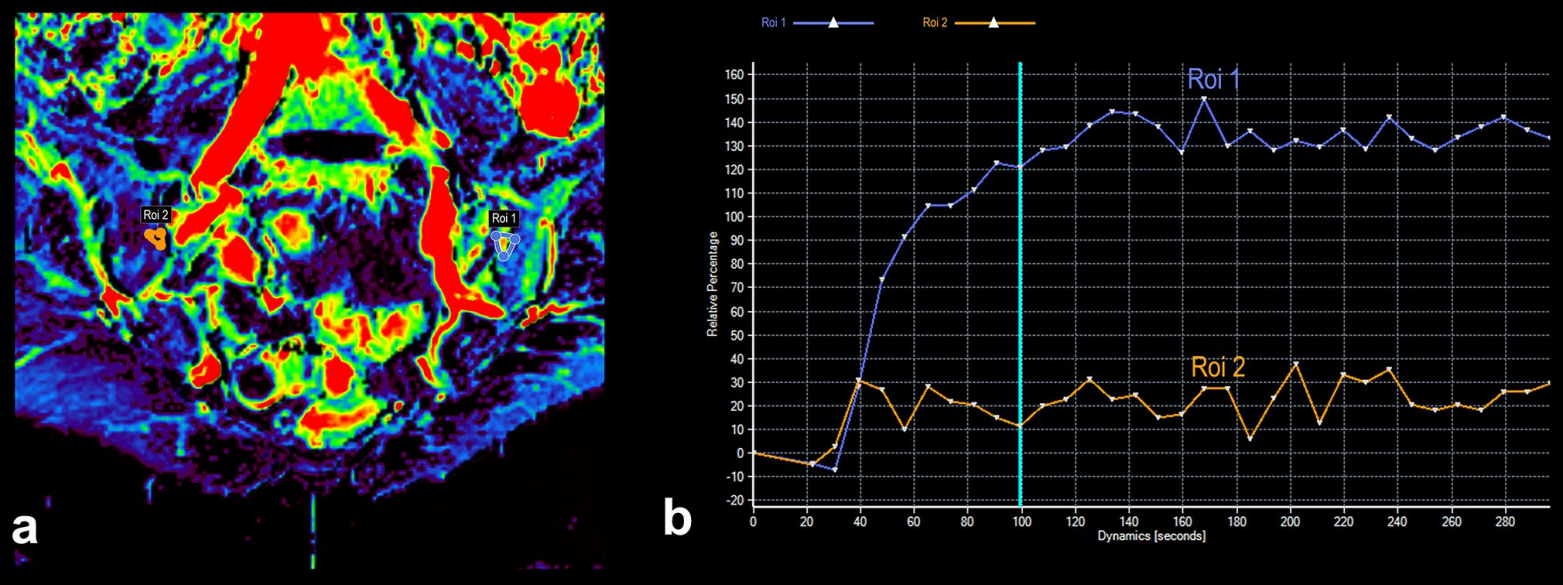
Area under the perfusion curve colour-coded map of the sacral region of the person with axSpA, with marked inflammatory lesion in the upper iliac quadrant of the left sacroiliac joint (Roi 1, blue line) and respective unaffected area in the right sacroiliac joint (Roi 2, orange line) (**a**). The graph of relative percentage enhancement versus time of acquisition showing the pattern of enhancement in typical axSpA active inflammatory lesion (Roi 1, blue line) in comparison to intact, non-enhancing tissue (Roi 2, orange line) (**b**)

All current guidelines regarding the diagnostics and monitoring of the axSpA with the use of MRI advise only the use of a few basic sequences (fat-suppressed T2-weighted, STIR, fat-suppressed contrast-enhanced T1-weighted sequences). Nevertheless, the advancement in the field of MRI imaging leads to the popularization of diffusion-weighted imaging (DWI) combined with apparent diffusion coefficient (ADC) maps, and dynamic contrast-enhanced (DCE) sequences, also in rheumatology. Example of these techniques’ application in the visualisation of active inflammatory lesions of patients with axSpA is shown in Figs. 2 and 3. However, utility of both these sequences in the assessment of axSpA arouses controversy for the time being. On one hand, the majority of the current studies describes these sequences as highly sensitive methods of early sacroiliitis diagnosis and its effective differentiation, additionally enabling quantitative assessment of inflammatory changes for purposes of the disease activity monitoring [22–25]. An example of such quantitative analysis of enhancement curve within the area of active inflammatory lesion compared to unaffected area based on DCE sequence is presented in Fig. 4. On the other hand, some authors question the beneficial value of incorporating these sequences to the basic image acquisition protocol used for diagnostics of axSpA [26]. Taking into account that up to this point, the literature on this topic is very limited, the use of DWI and DCE sequences in the diagnostics, differentiation, and quantitative monitoring of the axSpA seems to be the promising topic for future research. However, DWI appears to be more beneficial, because it is fast sequence which does not require gadolinium administration.

Another auspicious modality which is extensively investigated in rheumatology is whole-body MRI, which allows to comprehensively evaluate axial and peripheral articular inflammatory changes along with enthesitis [27–29]. The general assessment of the whole musculoskeletal system appears as the potentially effective tool of axSpA differentiation and detailed disease activity monitoring.

### Other imaging modalities

According to EULAR recommendations [1]:

1. Scintigraphy and ultrasound are not recommended for diagnosis of sacroiliitis as a part of axSpA.
2. Future research agenda: investigation of new/alternative imaging technologies which may be potentially useful for clinical purposes (ultrasound—new transducers, Doppler quantification, elastosonography; new nuclear medicine techniques; optical imaging).

At this moment, recommendations do not include ultrasonography as a sufficient method of axSpA-related sacroiliitis diagnosis and monitoring. As the ultrasonography is imaging method which is the most dependent on its operator experience, when combined with the anatomic complexity of this region, it significantly limits the daily usage of this method in axSpA diagnostics and management. Nonetheless, since it allows to visualise articular and soft-tissue periarticular involvement of the SIJs, it may be considered as one of the methods facilitating early axSpA diagnosis in less evident cases [30, 31]. Colour and duplex Doppler sonography with or without the use of specific contrast media may turn out as a perfect tool for long-term monitoring of the SIJs inflammation activity and response to treatment, as it is suggested in some preliminary reports [32, 33].

Moving to nuclear medicine techniques, bone scintigraphy was widely used for axSpA-related sacroiliitis detection in the past, thanks to the capability of illustrating the regions of inflammation and high bone turnover within the SIJs. Nevertheless, this technique was superseded by MRI, as a consequence of its low sensitivity, specificity, and accuracy [31]. Popularization of the hybrid imaging methods in rheumatology may be a new hope of early axSpA diagnosis. Up to this point, few studies focusing on the use of ^18^F-PET/MR and ^18^F-PET/CT were published. Although the data on the utility of these methods in the field of axSpA are sparse, yet they suggest that they may be advantageous in terms of detection of future new bone formation areas (visualisation of osteoblastic activity and hyperaemia) and assessment of disease activity [34–36].

## Assessment of the spine

### Conventional radiography

According to EULAR recommendations [1]:

1. CR of the spine might be used for long-term monitoring of structural damage in axSpA, especially new bone formation.
2. In patients with AS, initial CR of the lumbar and cervical spine is recommended to detect syndesmophytes, which are predictors of new syndesmophytes formation.

The role of the CR of the spine in fact is limited to the monitoring of late stage of axSpA progression, namely, AS, as only chronic changes, such as ankylosis and syndesmophytes, are visible there [4]. As far as semiquantitative methods of spinal structural changes scoring is concerned, the most popular and reliable one is the modified Stoke Ankylosing Spondylitis Spinal Score (mSASSS) [4]. This method of evaluation base on the assessment of anterior corners of cervical and lumbar spine vertebrae in the lateral view—from the lower border of C2 to the upper border of Th1 and from the lower border of Th12 to the upper border of S1 (including). Each corner is scored from the 0 to 3 points, depending on its morphology (0—normal; 1—sclerosis, squaring or erosion; 2—syndesmophyte; 3—bony bridge), and the maximal possible score is 72 points [4].

### Computed tomography

CT of the spine is not included in any set of recommendations as an advised method of the spine structural damage assessment. In general, it is understandable, taking into account the extremely high dose of ionizing radiation during CT examination of such large area as the spine and satisfactory performance of spine CR. Nonetheless, CR assessment, especially only with the use of the mSASSS score, omits evaluation of frequently affected by axSpA thoracic vertebrae as well as posterior vertebral corners and facet joints. In addition, the imprecise character of CR images hinders the short-term progression evaluation, as it may take up to 2 years to observe axSpA radiographic progression [13]. These drawbacks of CR lead to the development in the field of low-dose CT as a possible method of chronic bony changes assessment in the spine. This technique detects significantly more bony proliferation lesions than CR combined with the mSASSS scoring system [37].

Newly proposed CT Syndesmophyte Score (CTSS) seems to be a reliable tool for precise assessment of new bone formation in the course of AS with the use of low-dose CT [38]. The spine is analysed in two planes: sagittal and coronal, where the anterior + posterior rim and the right + left rim are assessed, respectively—in summary four quadrants. Every vertebra is additionally divided in upper and lower half, which are evaluated separately. The score takes into account the area from the bottom half of the C2 vertebra to the upper half of S1 vertebra (including). Every quadrant of vertebrae half is scored with 0 to 3 points—details concerning the rules of scoring are demonstrated in Table 2. Maximal possible score is 552 points [38]. This method, apart from the precise monitoring of AS progression, enables to quantitatively analyse the effect of biological therapy on new syndesmophytes formation.

**Table 2:** Scoring system of syndesmophytes according to the CT Syndesmophyte Score

Score	Description of changes
0	Syndesmophyte absent
1	Syndesmophyte reaches < 50% of the intervertebral disc space
2	Syndesmophyte reaches ≥ 50% of the intervertebral disc space, but does not form the bridge
3	Syndesmophyte bridge

### Magnetic resonance imaging

According to EULAR recommendations [1]:

1. MRI of the spine is not generally recommended in the diagnostics of axSpA.
2. MRI of the spine may be used to evaluate and monitor the axSpA activity, providing additional information to clinical and biochemical assessments.
3. MRI of the spine may be used to predict development of new, radiographic syndesmophytes—especially relevant are such changes as vertebral corner inflammatory or fatty lesions.
4. Extensive inflammatory activity, particularly on MRI of the spine of patients with AS, may be used as a predictor of good clinical response to anti-TNFα treatment in axSpA. As well, it may aid the decision of starting biologic therapy, in addition to clinical examination and CRP.

The principal active inflammatory lesion which is detected on MRI of the spine in patients with axSpA is bone-marrow oedema in the area adjacent to: anterior and posterior corners of vertebrae (spondylitis), vertebral endplates (spondylodiscitis), insertions of the anterior and posterior longitudinal ligaments (osteitis related to enthesitis), or facet and costovertebral joints (arthritis). As well, enthesitis of the spinal ligaments may be visible. Chronic inflammatory changes, which could appear in the course of axSpA, are: fat deposition on vertebral corners, erosions, syndesmophytes, and ankylosis [4, 39]. However, according to ASAS consensus (2012), it is the anterior and posterior spondylitis along with fatty depositions on vertebral corners which are lesions typical for axSpA. If anterior/posterior spondylitis is located in three or more sites in the spine and fatty depositions on several vertebral corners, they could be classified as highly suggestive of axSpA, especially in younger patients. The rest of inflammatory or structural changes are either non-specific or not sufficiently investigated yet to include them in final consensus [39]. Nonetheless, the assessment of MRI of the spine may only play supporting role in the diagnostics of the axSpA. The ASAS MRI working group did not recommend adding MRI of the spine into the definition of positive, suggestive of axSpA MRI as not beneficial for reaching the diagnosis, since the group of patients with positive MRI of the spine and negative MRI of the SIJ is marginal [16, 40]. Yet, it still could be a great method of disease activity and response to treatment monitoring.

Analogical to the one applying to MRI of the SIJs, there is a quantitative analysis method dedicated to the spine, called SPARCC spine inflammation score. Discovertebral units, namely, an intervertebral disc and adjacent endplates, are divided into four quadrants: lower anterior, lower posterior, upper anterior, and upper posterior endplates. If the increased signal is identified in particular quadrant, it receives 1 point, if not, 0 points. One additional point could be added for the presence of intense signal and another for the lesion ≥ 1 cm in depth. Each quadrant is assessed in three consecutive sagittal slices, which gives a maximal score of 18 per whole discovertebral unit. This method of evaluation should be applied to 6 affected discovertebral units, and therefore, the maximal possible score is 108 points. The whole analysis should be performed on fat-suppressed T2-weighted or STIR sequence [41].

Basic sequences used for the spine imaging in MR are: T1-weighted, fat-suppressed T2-weighted, STIR, and post-gadolinium fat-suppressed T1-weighted sequence [39]. The role of other sequences, such as DWI and DCE, is almost unexplored yet. There is only one report on this topic [42], which suggest that DWI is a sensitive and quick method of spinal active inflammatory changes’ evaluation. This remains another prospective branch which may significantly facilitate diagnosis and progression monitoring of axSpA in future.

## Conclusion

According to the EULAR and ASAS recommendations, conventional radiography and magnetic resonance imaging remain the basic methods of axSpA diagnosis, monitoring and response to treatment assessment. However, there is still a need to search for more accurate methods of early diagnosis and progression assessment, and alternative MRI sequences (DWI, DCE sequences), low-dose CT and hybrid imaging may be these missing links.
